# Erratum: The promise of neuroprotective agents in Parkinson's disease

**DOI:** 10.3389/fnins.2013.00069

**Published:** 2013-05-01

**Authors:** Stacey E. Seidl, Judith A. Potashkin

**Affiliations:** ^1^The Department of Biological Sciences, DePaul UniversityChicago, IL, USA; ^2^The Cellular and Molecular Pharmacology Department, The Chicago Medical School, RFUMSNorth Chicago, IL, USA

After our article was published online, it was brought to our attention that in Table 1 we incorrectly stated the findings from a study testing the safety of Isradipine (Simuni et al., 2010). We have thus changed the text in the table to reflect the results that Isradipine has been shown to be safe and well-tolerated in PD patients.

**Table 1:** **Neuroprotective Agents in PD models**.

**Neuroprotective agents**	**Cell culture and animal studies**		**Human and epidemiological studies**	
	**Study**	**Results**	**Study**	**Results**
Caffeine	A (Van den Pol, 1986; Kachroo et al., 2010)	Decreased dopaminergic neuron toxicity in MPTP	E (Ross et al., 2000; Ascherio et al., 2001)^*^, Saaksjarvi et al., 2008; Costa et al., 2010)^*^	Caffeinated beverages decreased the risk of PD
	A (Chen et al., 2001; Joghataie et al., 2004; Aguiar et al., 2006)	Decreased DA loss and restored DA levels in MPTP and 6-OHDA	E (Xu et al., 2006)	Caffeinated beverages have no effect on PD risk
	A (Joghataie et al., 2004; Aguiar et al., 2006)	Decreased motor dysfunctions in 6-OHDA	H (Ascherio et al., 2003)	A decrease in PD risk among women consuming caffeine and not taking hormone-replacement therapy
	A (Xu et al., 2002, 2010)	Caffeine increased metabolites associated with prevention of DA loss		
	A (Xu et al., 2006)	Estrogen and Caffeine prevented neuroprotection		
Caffeine + nicotine	A (Trinh et al., 2010)	Decaffeinated coffee and nicotine-free tobacco were neuroprotective in *Drosophila*	E (Tan et al., 2003)	Caffeine and nicotine combined reduced the rate of PD
Nicotine	A (Ferger et al., 1998; Costa et al., 2001)	Nicotine reduced DA depletion resulting from MPTP and 6-OHDA	E (Quick, 2004; Simon et al., 2009)	Nicotine lowered the risk of developing PD
	A (Meshul et al., 2002)	Nicotine minimized parkinsonian contralateral rotations in 6-OHDA		
	A (Quik et al., 2006)	Non-human primates maintained dopaminergic function and cell loss in the SNpc was prevented with nicotine administartion		
	A (Carr and Rowell, 1990)^*^	Tobacco smoke prior to MPTP treatment reduced the loss of striatal DA in mice		
Urate and UA	A/C (Jones et al., 2000; Duan et al., 2002)	UA protects against DA-induced apoptosis	E (de Lau et al., 2005, Annanmaki et al., 2007, Chen et al., 2009), Alonso et al., 2007)^*^, (Schwarzschild et al., 2008)^*^, (Andreadou et al., 2009)^*^	Decreased risk of PD with UA and urate
			H (Ascherio et al., 2009)	Slower rates of clinical progression of PD were seen with UA intake
			H (de Lau et al., 2005; Schwarzschild et al., 2008)	Serum UA correlates with a decreased risk of PD
Vitamin E	A (Odunze et al., 1990)	Vitamin E deficiency increases MPTP toxicity	E (Zhang et al., 2002; Etminan et al., 2005)^*^	Protection from PD with moderate vitamin E intake
	A (Lan and Jiang, 1997; Roghani and Behzadi, 2001)	Vitamin E supplimentation protected DA neurons in the SNpc and reduced DA loss	H (Fernandez-Calle et al., 1992; LeWitt, 1994; Morens et al., 1996)	Clinical trials also show no neuroprotective benefit of taking vitamin E
	A (Gong et al., 1991; Chi et al., 1992)	Striatal DA was not attenuated by pretreatment of vitamin E		
	C (Offen et al., 1996)	Vitamin E has no protective effects against DA-induced toxicity in PC12 cells		
	A (Perry et al., 1987)	Vitamin E partially protected DA neurons in MPTP rodents		
Vitamin E + vitamin C			H (Fahn, 1992)	Both vitamins combined decreased PD progression in early stage patients
Vitamin C			H (Scheider et al., 1997; Zhang et al., 2002; Etminan et al., 2005)	Few beneficial effects are seen with vitamin C and even an increased risk of PD
			H (Perlmutter, 1988)	Vitamin C intake reduced the risk of PD by 40%
Vitamin D	C (Butler et al., 2011)	Disruption of Vitamin D's Ca^2+^ homeostasis properties accelerated SNpc dopaminergic neuron loss	E (Anderson et al., 1999)	An increased risk of PD is associated with high consumption of vitamin D
	H/A (Gash et al., 1996; Kordower et al., 2000), (Gill et al., 2003)	GDNF stimulation by Vitamin D can alleviated PD symptoms in primates and PD patients		
	C/A (Wang et al., 2001; Smith et al., 2006)	Vitamin D produced beneficial effects against PD characteristics		
	A (Holick, 2007)	Vitamin D increased neuromuscular function in parkinsonian rodents		
Beta-carotene	(Perry et al., 1985, 1987; Yong et al., 1986)	Beta-carotene protected against MPTP neurotoxicity in mice, but not primates	E (Perlmutter, 1988)	A decrease in the risk of PD was seen with high B-carotene intake
Riboflavin			E (Perlmutter, 1988; Murakami et al., 2010)	Reduced risk of PD, with high raboflavin intake by 51%
			E (Coimbra and Junqueira, 2003)	Riboflavin supplimentation improved motor capacity of PD patients
CoQ10	A (Shults et al., 1999)	CoQ10 protected nigrostriatal dopaminergic neurons in MPTP	H (Shults et al., 2002; Shults, 2003; Galpern and Cudkowicz, 2007)	Chronic administration of CoQ10 delayed progression of PD in patients with no adverse affects in Phase II
	A (Cleren et al., 2008)	CoQ10 supplementation diminished neural tissue damage and prevented DA depletion in SNpc	Investigators (2007)	Inconclusive results of CoQ10 supplement showing neuroprotection in PD patients
CoQ10 + vitamin E			H (Shults et al., 2004)	Combination showed beneficial for PD patients, however, Phase III trials deemed it futile (NINDS, see text footnote 1)
Creatine	A (Matthews et al., 1999)	Creatine protected against MPTP-induced DA depletion in the SNpc	Investigators (2006)	Creatine delayed the progression of PD by 50%
			Investigators (2008)	Creatine showed efficacy as a neuroprotective agent in PD and is currently in Phase III trials (NET-PDLS, see text footnote 2)
CoQ10 + creatine	A/H (Yang et al., 2009)	Combination showed a neuroprotective effect in chronic MPTP and humans		
DHA	A (Bousquet et al., 2008)	DHA supplements replaced omega-6 fatty acids after MPTP		
	C (Wang et al., 2003)	Neurons were protected against cytotoxicity with DHA intake		
	A (Ozsoy et al., 2011)	DHA decreased apoptosis of dopaminergic cells in MPTP		
	A (Samadi et al., 2006)	DHA reduced 40% of the levodopa-induced dyskinesias in Parkinsonian primates		
	A (Bousquet et al., 2008)	DHA preserved DA levels from MPTP-induced neurotoxicity in mice		
DHA + uridine	A (Wurtman et al., 2006; Sakamoto et al., 2007)	This combination increased levels of neural phosphatides, proteins in synaptic membranes, and dendritic spines in rodents		
	A (Cansev et al., 2008)	DHA and uridine administration also reduced parkinsonian related behaviors and elevated DA levels in 6-OHDA rats		
Melatonin	A (Acuna-Castroviejo et al., 1997; Kim et al., 1998; Maharaj et al., 2006a)	Neuronal cell death damage induced by MPTP, 6-OHDA, and iron was protected with Melatonin administration	H (Dowling et al., 2005)	Melatonin improved duration of sleep and reduced sleep disturbances in PD patients
	A (Mayo et al., 1998, 1999)	Melatonin blocked apoptosis and necrosis in 6-OHDA and PC12 cells		
	A (Tapias et al., 2010)	Striatal DA depletion and DA neuron loss increased after melatonin treatment of rotenone-induced Parkinsonism		
GSH	A (Garrido et al., 2011)	Excessive or reduced GSH levels initiated degeneration of DA neurons		
	C (Schulz et al., 2000)	Decreased GSH increased neuron susceptibility to neurotoxins, but did not correlate to DA viability or striatal terminals		
	A (Klivenyi et al., 2000)	Depletion of DA was seen after MPTP treatment in GSH peroxidase-deficient mice		
	A (Pileblad et al., 1989; Seaton et al., 1996; Wullner et al., 1996)	Low levels of GSH reduced DA neurons after toxin administration		
IP6	A/C (Xu et al., 2008, 2011)	IP6 inhibited MPTP, 6-OHDA, and iron toxicity in cell culture		
	A (Xu et al., 2008)	IP6 increased cell survival in MPTP		
	A (Obata, 2003)	IP6 suppressed hydroxyl radical formation after MPP+ treatment in rats		
NSAID (ibuprofen+ aspirin)	A/C (Bilodeau et al., 1995; Kaufmann et al., 1997; Aubin et al., 1998; Saini et al., 1998; Casper et al., 2000; Sairam et al., 2003)	NSAID's protected against neuronal death and dopaminergic neurotoxicity	E (Chen et al., 2003; Ton et al., 2006; Wahner et al., 2007; Gao et al., 2011)	NSAID's lowered the risk of PD
			E (Chen et al., 2005; Ton et al., 2006; Driver et al. 2011, Gao et al., 2011)	A reduction in PD risk was observed with Ibuprofen, but not NSAIDS or Acetaminophen
	A/C (Esposito et al., 2007)	NSAIDS showed neuroprotection in MPTP, 6-OHDA, and *in vitro*	E (Becker et al., 2011)	NSAID's and aspirin showed no association with altering the risk of PD
	A (Casper et al., 2000, Morioka et al., 2004, Carrasco et al., 2005)	Ibuprofen protected DA neurons against glutamate toxicity and decreased MPTP toxicity	E (Bower et al., 2006; Hernan et al., 2006)	Increased risk of PD shown with moderate aspirin intake
			E (Samii et al., 2009, Seroka, 2010)	Ibuprofen reduced the risk of developing PD in humans by 40%
Isradipine	A (Ilijic et al., 2011)	Isradipine showed neuroprotection against 6-OHDA	H (Simuni et al., 2010)	Isradipine has been shown to be safe and well-tolerated in PD patients
Phenylbutyrate	C (Gardian et al., 2004; Zhou et al., 2011)	Phenylbutyrate protected DA neurons in the SNpc		
	A (Zhou et al., 2011)	Reduced deterioration in motor and cognitive function in mice		
Ex-4	A (Li et al., 2009)	Protected DA neuron degeneration, preserved DA levels, and improved motor function in rodents		
	A/C (Harkavyi et al., 2008; Li et al., 2009)	Ex-4 protected ventral mesencephalic dopaminergic cells in culture, reverse nigral lesions, and protected against 6-OHDA toxicity		
Ragasiline	C (Heikkila et al., 1985; Huang et al., 1999; Speiser et al., 1999; Maruyama et al., 2000; Sagi et al., 2001; Youdim et al., 2001a)	Reduces MPTP and 6-OHDA toxicity in PC12 and SH-SY5Y cells	H (Hauser et al., 2009; Olanow et al., 2009)	Rasagiline reduced the long-term progression and symptoms in PD
	A (Kupsch et al., 2001, Sagi et al., 2001)	Rasagiline prevented nigrostriatal damage induced by MPTP in primates		
	A (Blandini et al., 2004)	Rasagiline increased DA neuron survival in lesioned SNpc and improved motor impairments	H (Rascol et al., 2011)	Rasagiline in a Phase III delayed the need for antiparkinsonian drugs and patients had lower scores on the Parkinson's disease rating scale
	C (Murer et al., 2001)	Rasagiline increased expression of neurotrophins		
Minocycline	A/C (Du et al., 2001)	Minocycline blocked MPTP-induced degeneration of DA neurons in the SNpc~ preventing loss of striatal DA and its metabolites. Minocycline treatment also inhibited MPP+ mediated inducible NO synthase expression *in vivo* and blocked NO-induced neurotoxicity *in vitro*	H (Investigators, 2006)	Minocycline was deemed effective in Phase II slowing the progression of PD in patients. An 18-month follow up study showed no safety concerns with its use (Investigators, 2008), leading to Phase III trials
	A (Faust et al., 2009; Radad et al., 2010)	DA neuroprotection by Minocycline was seen in a *Drosophila* model of PD and after rotenone toxicity in rodents		
	A (Quintero et al., 2006)	Reduced the number of apomorphine-induced rotations in 6-OHDA-lesioned rats		
	A/C (Yang et al., 2003)	Minocycline exacerbated MPTP damage to DA neurons *in vitro* and *in vivo*		
	A (Diguet et al., 2004)	Minocycline treatment in primates and mice produced more severe/rapid parkinsonism, behavior deficits, and greater loss of nerve endings		
Minocycline + creatine			H (NET-PD, 2006)	Reduced progression in PD patients in Phase II

C, cell culture; A, animal; H, human; and E, epidemiological. Studies showing gender specificity, where males show favorable results are denoted (^*^).
